# Anti-Inflammatory and Anti-Nociceptive Activities of Methanol Extract from Aerial Part of *Phlomis younghusbandii* Mukerjee

**DOI:** 10.1371/journal.pone.0089149

**Published:** 2014-03-05

**Authors:** Qiu-Shi Wang, Li Yang, Wen-Yao Cui, Lei Chen, Yan-Hua Jiang

**Affiliations:** 1 Anesthesiology Department of the First Affiliated Hospital of China Medical University, Shenyang, China; 2 Department of Pharmacology of Liaoning University of Traditional Chinese Medicine, Shenyang, China; 3 Department of Pharmacology of China Medical University, Shenyang, China; University of Milan, Italy

## Abstract

This study was designed to investigate the anti-inflammatory and anti-nociceptive activity of the methanol extract from the aerial part of *Phlomis younghusbandii* (MEAP) and to explore the possible related mechanisms. Anti-inflammatory effects of MEAP were evaluated by using the ear edema test induced by dimethylbenzene and vascular permeability test induced by acetic acid. Anti-nociceptive activities of MEAP were evaluated by the chemical nociception in models of acetic acid-induced writhing and formalin-induced hind paw licking, and by the thermal nociception in hot plate tests. Mechanisms of MEAP activities also were explored by evaluating expression levels of TNF-α, IL-6 and iNOS induced by LPS using real-time fluorogenic PCR and expression of COX-2 using Western blotting and an open-field test. The results indicated that the MEAP administered orally could significantly decrease ear edema induced by dimethylbenzene and increase vascular permeability induced by acetic acid. Additionally, the nociceptions induced by acetic acid and formalin were significantly inhibited. The anti-nociceptive effect could not be decreased by naloxone in the formalin test, and MEAP did not affect the normal autonomic activities of mice. Expression levels of pro-inflammatory cytokines (TNF-α, IL-6, iNOS) induced by LPS were decreased obviously by treatment with MEAP. Furthermore, COX-2 expression in the spinal dorsal horns of the pain model mice induced by formalin was significantly down-regulated by MEAP. In conclusion, MEAP has significant anti-inflammatory and antinociceptive activities, and the mechanisms may be related to the down-regulated expression of TNF-α, IL-6, iNOS and COX-2.

## Introduction

Inflammation, which is characterized by pain, redness, swelling and dysfunction of the tissues and organs, is the normal result of host protective responses to tissue injury caused by numerous stimuli (e.g., physical trauma, chemicals and infectious agents) [1]–[4]. Inflammation is commonly associated with pain as a secondary process, resulting from the secretion of analgesic mediators [5]. To protect against outer stimuli or tissue injury, various pro-inflammatory mediators, including tumor necrosis factor alpha (TNF-α), interleukin-6 (IL-6) and nitric oxide (NO), are released by the host cellular immune response system [6]. However, the excessive release of pro-inflammatory mediators may activate the inflammatory cascade reaction, leading to systemic inflammatory response syndrome (SIRS) [7]. In addition, prostaglandin E2 (PGE2), a major pain enhancing inflammatory mediator, can be induced by cyclooxygenase 2 (COX-2) in the process of inflammation. Previous investigations have demonstrated that it is beneficial for treating inflammatory diseases to down-regulate the expression of TNF-α, IL-6 and COX-2 [1], [2], [8].

*Phlomis younghusbandii* Mukerjee, a perennial herbal plant belonging to the genus *Phlomis* of the family Lamiaceae, is only distributed in the eastern region of the Qinghai-Tibet Tibetan Plateau in China [9]. The root of *P. younghusbandii* has been traditionally used in Tibetan medicine as an important crude drug to treat anemopyretic cold, cough with profuse sputum, throat inflammation, skin infection, bronchitis and pneumonia [9], [10]. Recently, the root extracts of this plant have been reported to relieve cough and expell phlegm [11], as well as demonstrated anti-inflammatory, anti-nociceptive [12] and anti-bacterial [13] pharmacological activities. Previous phytochemical investigations on *P. younghusbandii* showed the presence of iridoids [14]–[16], diterpenes [17] and flavones [16] in this plant. However, no chemical components or pharmacological activities of the aerial part of this plant have been reported thus far, because it is not used traditionally. In recent years, much effort has been placed towards not only utilizing the aerial part as a value-added product, but also potentially as a substitute for the root [18], [19].

In our previous work, the methanol extract of the aerial part of *P. younghusbandii* (MEAP) was found in preliminary experiments to have obvious anti-inflammatory and anti-nociceptive activities. Therefore, in the present study, we further investigated these pharmacological activities and underlying mechanisms of MEAP *in vivo* and *in vitro*, which would support its potential application as a preventive or therapeutic agent for disease.

## Materials and Methods

### Plant Material

The aerial part of *P. younghusbandii* was purchased from Hehuachi Market of Traditional Chinese Herbs in 2010 and identified by the department of Traditional Chinese Medicine in our hospital. A voucher specimen of *P. younghusbandii* (S201009-PY) was kept in our lab for future reference.

### Drugs and Chemicals

Methanol (AR), formalin (AR) and dimethylbenzene were purchased from Sinopharm Chemical Reagent Co., Ltd. (Shanghai, China). Morphine and naloxone were supplied by the pharmaceutical preparation section of our hospital. Indometacin was purchased from Sigma Chemical Co. (St. Louis, MO, USA).

### Ethics Statement

All animal treatments were conducted strictly in accordance with international ethical guidelines and the National Institutes of Health Guide concerning the Care and Use of Laboratory Animals. The experiments were carried out with the approval of the Animal Experimentation Ethics Committee of the First Affiliated Hospital of China Medical University.

### Animals

Experimental ICR mice (20±2 g) were obtained from the Shanghai Laboratory Animal Center (Shanghai, China). They were housed at 21±1°C under a 12 h light/12 h dark cycle and had free access to standard pellet diet (Purina chow) and tap water. Each animal was used only once in the experiment. For evaluation of the anti-inflammatory and anti-nociceptive activities of MEAP *in vivo*, 60 mice were randomly divided into 6 groups (n = 10), including the control, positive control and MEAP treatments (50, 100, 200 and 400 mg/kg). For evaluation of the toxicity of MEAP, 90 mice were randomly divided into 9 groups (n = 10) and treated with MEAP at various doses of 0, 25, 50, 100, 200, 400, 800, 1600 or 3200 mg/kg.

### Preparation of Test Sample

The dried and powdered aerial part of *P. younghusbandii* (500 g) was extracted under reflux three times (each extraction period lasted 3 h) and six times with methanol. The combined solution was centrifuged (10000× *g* for 10 min) and concentrated under reduced pressure to obtain a residue (30 g). The final obtained sample was used as the MEAP in our present investigations.

### High Performance Liquid Chromatography (HPLC) Analysis

HPLC was performed on an Agilent 1100 HPLC system (Agilent 1100, Germany), equipped with a quaternary pump, a diode array detector (DAD) and a computer with workstation software program for data analysis. The mobile phase for the HPLC was MeOH: dd Water (70∶30, v/v), and the detection wavelength was set at 248 nm. The injection volume was 10 µL, and the flow rate was 1 mL/min. A Thermo ODS Hypersil (250×4.6 mm i.d.; 5 µm) column was used set at the temperature of 30°C.

### Toxicity Tests

Ninety ICR mice were randomly divided into nine groups, with each group containing ten mice. Mice of groups 1–8 were orally administered 25, 50, 100, 200, 400, 800, 1600 and 3200 mg/kg of MEAP, respectively. Additionally, the 9^th^ group received 15 mL/kg of normal saline. The mortality rates of the mice within a 24 h period were observed and recorded.

### Assessment of Dimethylbenzene-induced Edema in Mice

Dimethylbenzene-induced ear edema in mice, produced as described by He *et al.* [20], was used to evaluate the anti-inflammatory activity of MEAP. Briefly, MEAP was administered orally 1 h before dimethylbenzene topical application to the right ear. Subsequently, mice were sacrificed under anesthesia with sodium pentobarbital (40 mg/kg, ip) at 1 h after dimethylbenzene treatment, and the ear edema was measured by subtracting the weight of the left ear from that of the right. The inhibition was calculated as follows: inhibition = (A–B)×100/A, where edema A is edema induced by dimethylbenzene alone, and edema B is edema induced by dimethylbenzene plus sample.

### Assessment of Acetic Acid-induced Vascular Permeability in Mice

The vascular permeability test in mice was performed according to the method described by He *et al.* [20]. Briefly, 0.7% (v/v) acetic acid in saline (10 mL/kg) was injected abdominally at 1 h after the administration of the MEAP. Simultaneously, 2% (w/v) Evans Blue (10 mL/kg) was injected intravenously. After 20 min, the mice were sacrificed under anesthesia with sodium pentobarbital (40 mg/kg, i.p.), and then the pigment that had leaked into the abdominal cavity of each mouse was rinsed with 5 mL of normal saline solution. The recovered wash solution was centrifuged at 780× g for 15 min, and the absorbance of the supernatant was measured at a wavelength of 630 nm.

### Cell Culture and Cell Viability Assay

RAW264.7 cells, a murine macrophage cell line, were cultured at a density of 4×10^5^ in plastic dishes containing Dulbecco's modified Eagle's medium (DMEM) 18 supplemented with 10% heat-inactivated fetal bovine serum (FBS). After pre-incubation for 24 h in a CO_2_ incubator (5% CO_2_) at 37°C, the RAW264.7 cells were cultured with MEAP (0, 10, 25, 50, 100, 200, 400 µg/mL) in the presence of 100 ng/mL lipopolysaccharide (LPS) for 24 h at 37°C. Thereafter, the cells were washed twice with phosphate-buffered saline (PBS) and incubated with 100 µL of 0.5 mg/mL MTT for 2 h to measure the cell viability. The medium was then discarded, and 100 µL dimethyl sulfoxide (DMSO) was added. After a 30-min incubation, absorbance at 570 nm was read using a microplate reader.

### Real-time Fluorogenic PCR Assays of Gene Expression of Pro-inflammatory Cytokines

RAW 264.7 cells were harvested, and total RNA was extracted using Trizol reagent (Carlsbad, CA, USA). Total RNA was used for cDNA synthesis of TNF-α, IL-6, iNOS and β-actin by reverse transcription using quantitative real-time PCR (Bio-Rad, Hercules, CA, USA). All mRNA primers were designed by Primer Premier 5.0 and synthesized by SBS Genetech Co., Ltd (Beijing, China). Primers used for the real-time PCR are shown in Table 1. Reverse transcription was performed according to the manufacturer's recommendation of the quantitative real-time RT-PCR reaction kit 11 (TOYOBO, Osaka, Japan).

**Table 1:** Primers Used for Real-time PCR.

Gene name		Primer sequence
TNF-α		F: 5′-CAGGTTCTGTCCCTTTCACTCACT-3′
		R: 5′-GTTCAGTAGACAGAAGAGCGTGGT-3′
IL-6		F: 5′-TGGAGTACCATAGCTACCTGGAGT-3′
		R: 5′-TCCT-TAGCCACTCCTTCTGTGACT-3′
iNOS		F: 5′-TCCTACACCACACCAAAC-3′
		R: 5′-CTCCAATCTCTGCCTATCC-3′
β-actin		F: 5′-GGGAAATCGTGCGTGACATCAAAG-3′
		R: 5′-CATACCCAAGAAGGAAGGCTGGAA-3′

F: Forward primer, R: Reverse primer.

### Abdominal Constriction Induced by Acetic Acid

The acetic acid-induced writhing test was determined according to a previously reported method with some modifications [21]. Mice were treated with MEAP orally and the vehicle 1 h before intraperitoneal (i.p.) administration of acetic acid (0.75%, 10 ml/kg). Indometacin was administered abdominally at the dose of 10 mg/kg 1 h before the experiment as a positive control. The number of writhes was counted for each animal, starting 3 min after the acetic acid injection over a period of 12 min.

### Hot Plate Test

Pain reflexes in response to a thermal stimulus were measured by the hot plate test and were performed according to a previously described method with some modifications [22]. A hot plate apparatus (model HZ66-ZH-YLS-6B, China) maintained at 55±1°C was used. Only mice that showed initial nociceptive responses between 5 and 30 s were selected for the experiment. The latency to the first sign of hind paw licking or jumping to avoid heat nociception was taken as the index of nociceptive threshold. In this test, pretreatment latencies were determined three times with 20 min intervals. Measurements were started 30 min after treatment of the animals with MEAP or the vehicle. A group of mice treated subcutaneously (s.c.) with morphine dissolved in physiological saline (10 mg/kg, 30 min before the test) was used as a positive control. The cut-off time was 60 s in the hot plate test in order to minimize skin damage. The vehicle used alone had no effect on nociceptive responses.

### Formalin Test

The formalin test was conducted as described previously [22], and mice were administered MEAP or vehicle. One hour later, each mouse was given 20 µL of 5% formalin (subplantar) in the right hind-paw. The duration of paw lickings as the index of painful response was determined at 0–5 min (early phase, neurogenic pain) and 20–25 min (late phase, inflammatory pain) after formalin injection. Morphine was used as a positive control drug, which was administered at the dose of 10 mg/kg, s.c., 30 min before the test. In order to verify the possible mechanism of MEAP, anti-nociception (400 mg/kg) mouse groups pretreated with naloxone were used. Naloxone was administered 15 min before the treatment of MEAP or morphine. Morphine and naloxone were dissolved in physiological saline. The vehicles used alone had no effect on nociceptive responses.

### Open-field Test

Effects of MEAP on spontaneous locomotor activity and exploratory behavior were assessed by the open-field test according to a method described previously [23], [24].

### Western Blot Analysis of COX-2 Expression

After the formalin test, mice were sacrificed under anesthesia with sodium pentobarbital (40 mg/kg, i.p.), and the spinal dorsal horn was collected on an ice and salt mixture. Total proteins of spinal cord tissue were extracted, and then equal amounts of protein (60 µg) were separated by sodium dodecyl sulfate/polyacrylamide gel electrophoresis (SDS/PAGE), blotted on polyvinylidene difluoride (PVDF) and probed with an anti-COX-2 rabbit monoclonal IgG (Wuhan Boster Bio-engineering Ltd. Co., Wuhan, China), followed by incubation with a goat anti-rabbit/HRP conjugate and chemiluminescence detection. Images were scanned with a Densitometer Scanner (GS800, Bio-Rad), and optical density (OD) values were analyzed using Quantity One software (Bio-Rad). To normalize for protein loading, antibodies directed against β-actin were used, and the COX-2 expression level was expressed as a relative value to that of β-actin.

### Statistical Analysis

All results are expressed as the mean ± standard error of the mean (SEM). Statistical analyses were performed using the two-tailed Student's t-test with a significance level of *P*<0.05.

## Results

### HPLC Analysis

**Figure 1:**
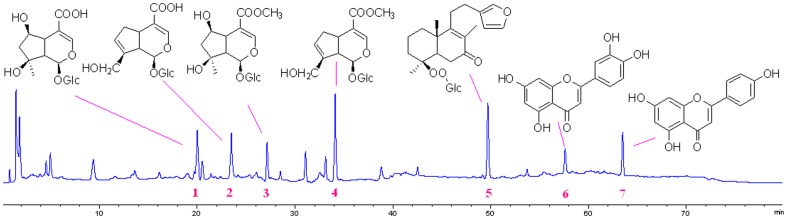
Chromatograms of MEAP and structures of the seven major components of MEAP. 1–7: shanzhiside, geniposidic acid, shanzhisidemethyl ester, geniposide, phlomisoside F, luteolin and apigenin.

Using our established HPLC conditions, chromatograms of MEAP were obtained (Fig. 1). Seven compounds, including shanzhiside, geniposidic acid, shanzhisidemethyl ester, geniposide, phlomisoside F, luteolin and apigenin, were identified as the major components ofMEAP by comparing individual peak retention times with those of the authentic reference standards. The chemical structures of these seven compounds are shown in Fig. 1.

### Toxicity Study Results

In the toxicity study, neither death nor any abnormal neuro-behaviors were observed during our test period. Thus, the 50% lethal dose (LD_50_) was not obtained due to lack of observable toxicity of the MEAP at any of the tested doses.

### Dimethylbenzene-induced Edema Test Results

**Figure 2:**
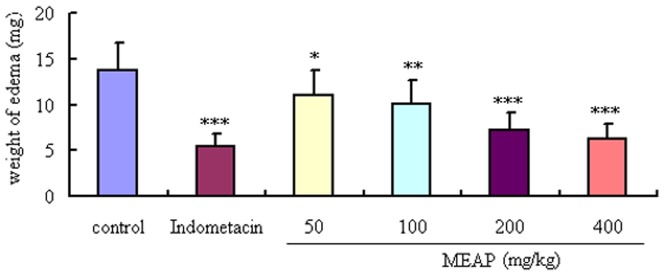
Effects of MEAP and indometacin on ear edema induced by dimethylbenzene in mice. The vehicle (control, 10 ml/kg) or the MEAP (400, 200, 100 and 50 mg/kg) was administered orally, and the indometacin (10 mg/kg) was given abdominally 1 h separately before each dimethylbenzene topical application to the right ear. Each column represents the mean ± SEM (n = 10). *P<0.05, **P<0.01, ***P<0.001, compared with control.

As shown in Fig. 2, the topical anti-inflammatory effect of MEAP was evaluated by measuring its inhibition of dimethylbenzene-induced ear edema in mice. MEAP showed significant dose-dependent effects on ear edema at 50, 100, 200 and 400 mg/kg with inhibitory rates of 19.56% (*P*<0.05), 26.47% (*P*<0.01), 47.42% (*P*<0.001) and 54.25% (*P*<0.001), respectively. At the 400 mg/kg dose, the anti-inflammatory activity of MEAP was comparable to indometacin at the dose of 10 mg/kg (54.25% vs. 60.58%). The observed topical anti-inflammatory properties of the MEAP indicated that it may be used to treat inflammatory diseases.

### Acetic Acid-induced Vascular Permeability Test Results

**Figure 3:**
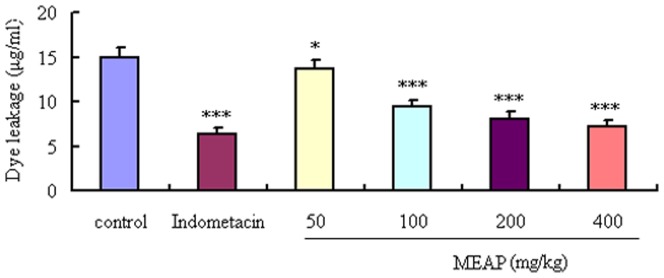
Effects of MEAP and indometacin (10 mg/kg) on leakage of dye into the peritoneal cavity. The vehicle (control, 10 ml/kg) or MEAP (400, 200, 100 and 50 mg/kg) was administered orally, and indometacin (10 mg/kg) was given abdominally. Each column represents the mean ± SEM (n = 10). *P<0.05, ***P<0.001, compared with control.

As shown in Fig. 3, MEAP at 50, 100, 200 and 400 mg/kg exhibited significant and dose-dependent inhibitory rates of 7.98% (*P*<0.05), 37.2% (*P*<0.001), 45.91% (*P*<0.001) and 52.21% (*P*<0.001) on the increased vascular permeability induced by acetic acid in mice. The positive control drug, indometacin (10 mg/kg, i.p.), also reduced the dye leakage considerably with an inhibition rate of 57.51%, similar to that of the MEAP at the dose of 400 mg/kg (*P*<0.001).

### Effects of MEAP on LPS-induced Cell Viability and Expression of TNF-α, IL-6 and iNOS

**Figure 4:**
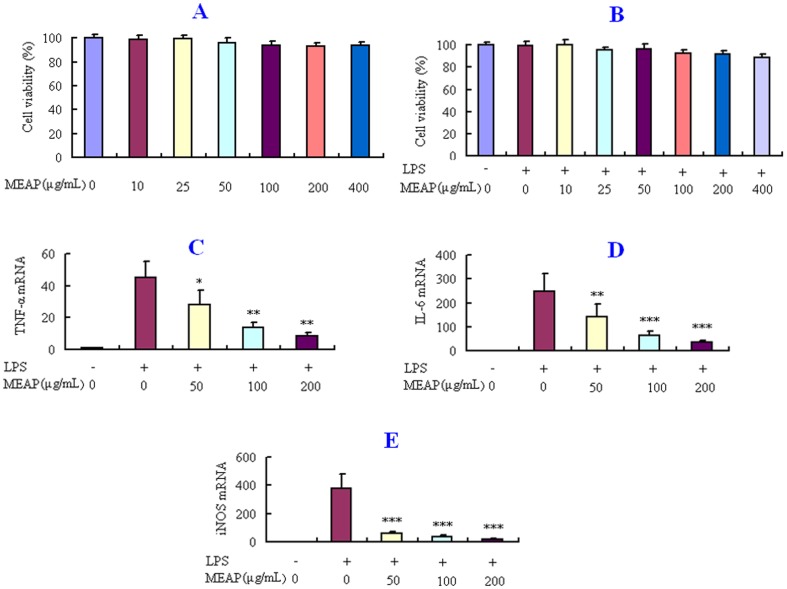
Effects of MEAP on TNF-α, IL-6, and iNOS mRNA expression in RAW 264.7 cells induced by LPS (100 ng/mL). TNF-α, IL-6, and iNOS mRNA expression levels were normalized by β-actin, and fold changes in mRNA expression are shown as the mean ± SEM (n = 4). *P<0.05, **P<0.01, ***P<0.001, compared with LPS alone.

Pro-inflammatory cytokines, such as TNF-α, IL-6 and iNOS, play important roles in the inflammatory process. To further validate the anti-inflammatory effects of MEAP, mRNA expression levels of TNF-α, IL-6 and iNOS induced by LPS were determined. The results showed that MEAP, at doses of 50, 100 and 200 µg/mL, could suppress significantly the expression of TNF-α, IL-6 and iNOS induced by LPS in RAW 264.7 12 cells (*P*<0.05) (Fig. 4C–E). To exclude the possibility that the inhibitory effects of MEAP on the production of pro-inflammatory cytokines mentioned above were due to cytotoxicity of MEAP, the MTT assay was performed. Whether the RAW 264.7 cells were treated with MEAP alone or with MEAP and LPS, there was no obvious change in cell viability (Fig. 4A, B).

### Acetic Acid-induced Writhing Test Results

**Figure 5:**
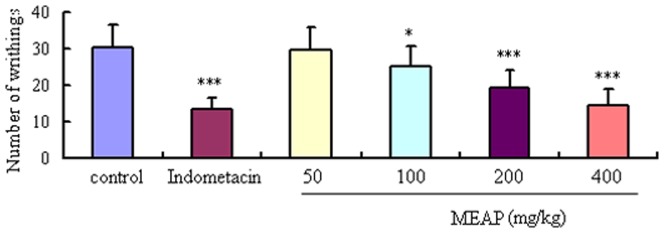
Effects of MEAP on acetic acid-induced writhing responses in mice. The vehicle (control, 10 ml/kg) or the MEAP (400, 200, 100 and 50 mg/kg) was administered orally, and indometacin (10 mg/kg) was given abdominally. Each column represents the mean ± SEM (n = 10). *P<0.05, ***P<0.001, compared with control.

As seen in Fig. 5, MEAP showed significant anti-noceceptive activities at doses of 100–400 mg/kg. The number of acetic acid-induced writhes decreased significantly at the test doses of 100–400 mg/kg, with inhibitory rates of 16.23, 36.42, and 51.66%, respectively. The positive drug, indometacin (10 mg/kg), also manifested significant antinociceptive activity with the inhibitory rate of 55.63%.

### Hot Plate Test Results

**Figure 6:**
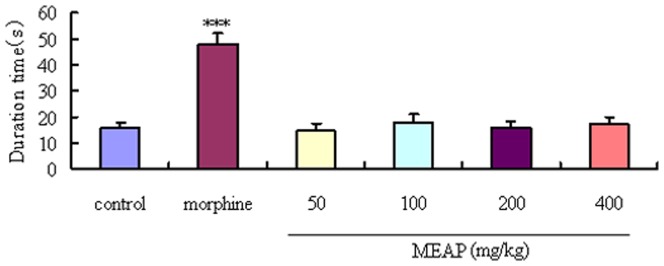
Effects of MEAP on thermal-induced nociception. The vehicle (control, 10 ml/kg) and MEAP (50, 100, 200, 400 mg/kg) were administered orally, and morphine (10 mg/kg) was given s.c. MEAP was administered 1 h before and morphine 30 min before the test. Each column represents the mean ± SEM (n = 10). ***P<0.001, compared with control.

Anti-nociceptive activities of MEAP in the pain model induced with a hot plate are shown in Fig. 6. No obvious anti-nociceptive effect of MEAP at the tested doses (50, 4 100, 200, 400 mg/kg) was observed. In contrast, the positive drug (morphine) manifested potent anti-nociceptive effects on the thermal nociception.

### Formalin Test Results

**Figure 7:**
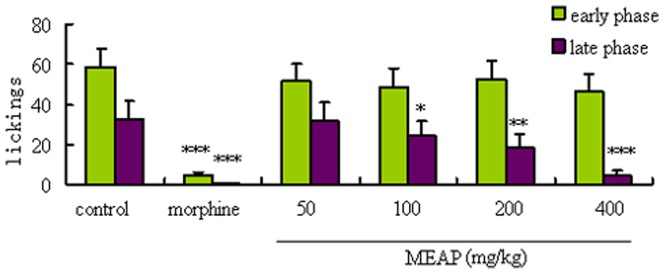
Effects of MEAP and morphine on formalin-induced nociception in mice. The vehicle (control, 10 ml/kg) and MEAP (50, 100, 200, 400 mg/kg) were administered orally, and morphine (10 mg/kg) was given s.c. MEAP was administered 1 h before the test. Each column represents the mean ± SEM (n = 10). *P<0.05, **P<0.01, ***P<0.001, compared with control.

**Figure 8:**
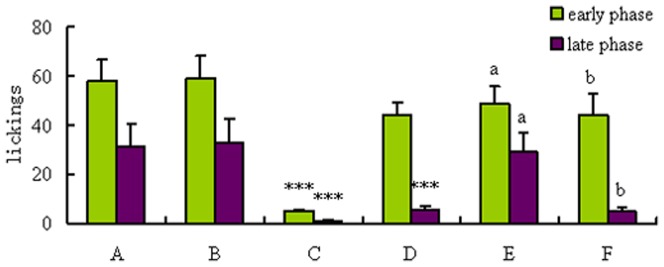
Effects of MEAP and morphine on formalin-induced nociception in mice in the formalin antagonism test. A: control, B: naloxone, C: morphine, D: MEAP (400 mg/kg), E: morphine + naloxone, F: MEAP + naloxone. The vehicle (control, 10 ml/kg) and the MEAP (400 mg/kg) were administered orally, and morphine (10 mg/kg) was given s.c. MEAP was administered 1 h before the test, and the naloxone (1 mg/kg, s.c.) was administered 15 min before the MEAP or morphine. Each column represents the mean ± SEM (n = 10). *P<0.05 and ***P<0.001, compared with control; ^a^ *P*<0.001, compared with morphine; ^b^ *P*>0.05, compared with MEAP.

Results of the anti-nociceptive effects of MEAP on pain induced by formalin in mice are shown in Figs. 7 and 8. Treatment with MEAP caused obvious diminutions of the late phase pain responses induced by formalin, in a dose-dependent manner (100, 200, and 400 mg/kg) (Fig. 7). In addition, the positive control agent, morphine (10 mg/kg), also showed powerful suppressive effects in both phases. When used alone, naloxone (1 mg/kg, s.c.) failed to modify the formalin induced nociceptive responses in a significant manner. In the combination investigations, naloxone could significantly antagonize the analgesic effect of morphine but failed to change the anti-nociceptive effects produced by MEAP (Fig. 8).

### Open-field Test Results

In our present study, the administration of MEAP (50, 100, 200, 400 mg/kg) was not able to obviously affect the locomotor activities of mice compared to the control group. The mean permanence time of mice and the length of the route obtained from the mice treated with MEAP were not statistically different from that of control group over a 5-min period. Only diazepam (1 mg/kg, i.p.) significantly (*P*<0.01) affected the mobile performance in comparison with the control group (data not shown).

### Western Blotting Results

**Figure 9:**
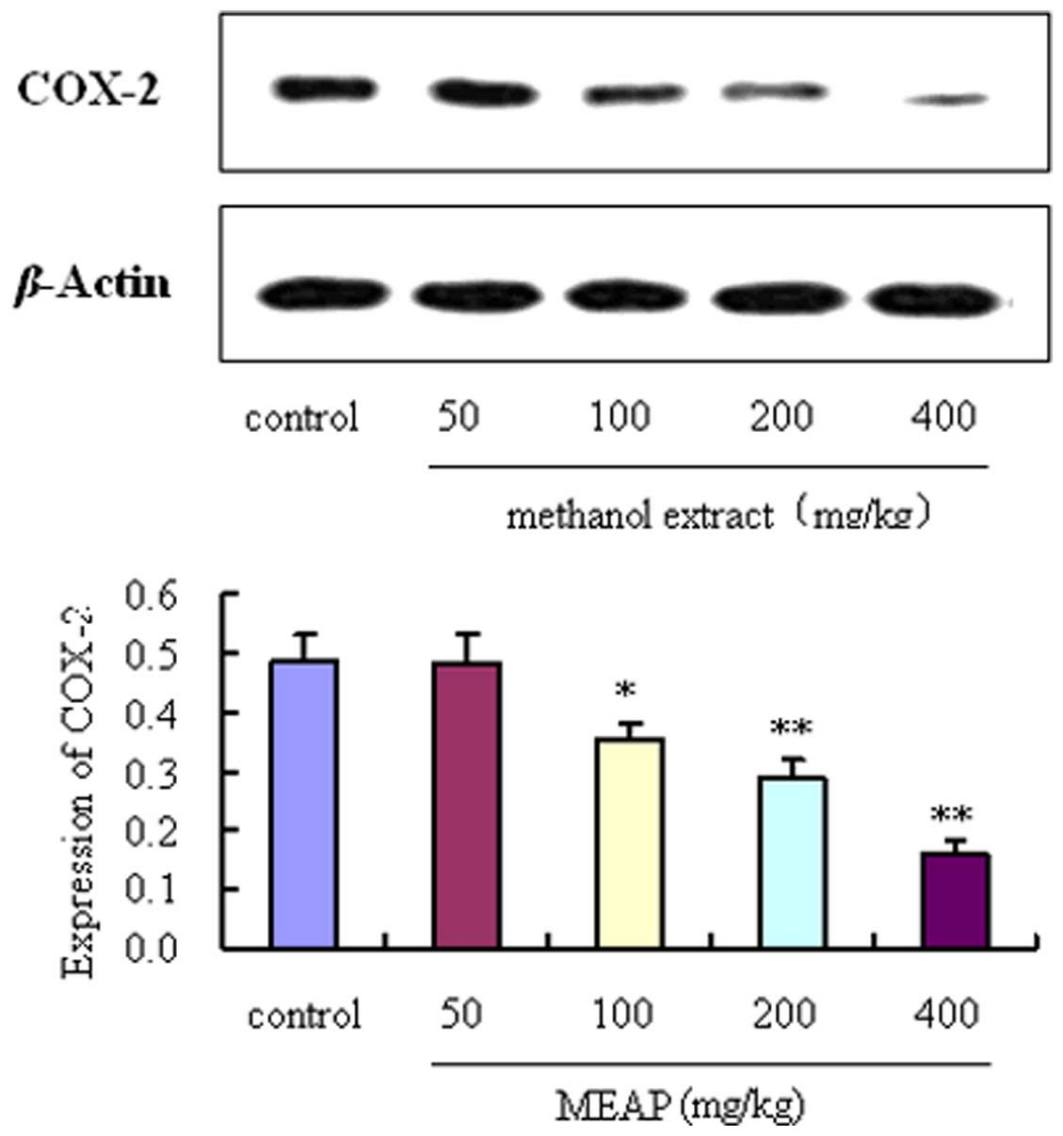
Effect of MEAP on expression of COX-2. Each column represents the mean ± SEM (n = 4). *P<0.05; **P<0.01, compared with control.

As shown in Fig. 9, expression levels of COX-2 in the spinal dorsal horns of mice treated with MEAP (100, 200 and 400 mg/kg) were significantly down-regulated compared with mice of the control group (*P*<0.05, *P*<0.01, *P*<0.01, respectively). Furthermore, MEAP showed obvious dose-dependent (100, 200 and 400 mg/kg) inhibitory effects on expression of COX-2 in the spinal dorsal horns of pain model mice induced by formalin (Fig. 9).

## Discussion

From time immemorial, natural products derived from plants have been used in folk medicines to prevent or treat various diseases worldwide. Plant-derived medicines are known to exhibit a wide range of dependable pharmacological activities [25]–[28]. In a previous investigation, the root of *P. younghusbandii* showed analgesic and anti-inflammatory activities [12], but no active constituent was reported. Compounds from the roots of *P. younghusbandii* or the genus of *Phlomis* are mainly iridoids, flavones, diterpenes and phenylethanoids [14]–[17], [21], [22]. In our present study, components of MEAP of *P. younghusbandii* were identified as iridoids (shanzhiside, geniposidic acid, shanzhisidemethyl ester and geniposide), diterpenes (phlomisoside F) and flavones (luteolin and apigenin). Thus, the major constituents of the aerial part of *P. younghusbandii* in our finding are consistent with the roots. Moreover, iridoids are reported to possess significant anti-inflammatory and anti-nociceptive activities [23], [29]. Based on the HPLC analysis, we can conclude that iridoids in MEAP may contribute to its anti-inflammatory and anti-nociceptive activities.

The dimethylbenzene-induced ear edema in mice is a preliminary and simple model for screening potential anti-inflammatory drugs [20]. In our study, MEAP showed a significant topical anti-inflammatory activity. However, assessment by the dimethylbenzene-induced ear edema model alone cannot be used to ascertain the anti-inflammatory effects of MEAP. Increased vascular permeability is an early and important vascular event in the inflammatory response [30]. Thus, the vascular permeability test was carried out to further demonstrate the anti-inflammatory effects of MEAP, which exhibited significant inhibitory effects on the increased vascular permeability in mice. Results from all of the animal model tests for anti-inflammation demonstrated the notable dose-dependent anti-inflammatory activities of MEAP, with the highest activity exhibited at the dose of 400 mg/kg. Furthermore, inhibition of the release of inflammatory cytokines and mediators can serve as a potential method for treatment of inflammatory diseases. In our investigations, MEAP was found also to significantly inhibit the mRNA expression of pro-inflammatory cytokines (TNF-α, IL-6 and iNOS) induced by LPS in RAW 264.7 cells, which is another line of evidence supporting the anti-inflammatory effects of MEAP.

The acetic acid-induced writhing test is the most useful model for preliminary studies of anti-nociceptive activity [23]. Intraperitoneal injections of acetic acid induces nociception though activation of chemosensitive nociceptors, or visceral surface irritation, leading to release of inflammatory mediators, including histamine, bradykinin, prostaglandin and serotonin [31]. In our study, MEAP showed significant anti-nociceptive activities in a dose-dependent manner in the acetic acid-induced writhing tests in mice. In addition, the acetic acid-induced writhing test is commonly used for evaluation of peripheral anti-nociceptive activity of drugs. Acetic acid can indirectly induce the release of endogenous mediators and stimulate the nociceptive neurons that are sensitive to non-steroidal anti-inflammatory drugs (NSAIDs) [32].

The hot plate test is widely used for evaluating central anti-nociceptive activities [33]. Results of the hot plate test in our present study indicated that MEAP is not a centrally acting analgesic. Therefore, we presumed that the MEAP exerts peripheral anti-nociceptive activities and performed the formalin test to confirm the hypothesis. The formalin test is a pain model consisting of two different phases, which can be separated in time. The early phase is generated peripherally through the activation of nociceptive neurons by the direct action of formalin, and the late phase refers to the inflammatory pain response involving the release of molecules such as prostaglandin, histamine and serotonin [34]. In our present study, the MEAP mainly suppressed pain of the late phase, which suggested that the anti-nociceptive mechanism of MEAP is related to the peripheral anti-nociceptive activity. This result was confirmed by testing MEAP in combination with naloxone (antagonist of morphine). By using the open-field test, a behavior model used to study exploratory and motor activity, we observed no obvious changes of locomotor activity (related to the central nervous system), which provided another piece of solid evidence to support the peripheral anti-nociceptive mechanism of MEAP activity.

Cyclooxygenase (COX) is the key enzyme in the synthesis of prostaglandins, and COX-2, an inducible enzyme, is responsible for the production of the pro-inflammatory prostaglandins [35]. Many former investigations have shown the important role of COX-2 in the induction of pain and inflammation as well as the analgesic actions of NSAIDs. In addition, COX-2 can induce synthesis of PGE, and the PGE_2_ released in inflamed tissues sensitizes the terminals of afferent nerve fibers, thereby enhancing nociceptive processing within the spinal cord and brain to evoke hyperalgesia [36]. Thus, one apparently feasible approach against nociception is to suppress the release of COX-2. In our study, MEAP significantly and dose-dependently inhibited expression of COX-2 in the spinal dorsal horns of pain model mice induced by formalin, which indicated that MEAP may be a potent COX-2 inhibitor.

## Conclusions

In conclusion, the experimental evidence acquired in our present study indicated that administration of MEAP can significantly suppress the ear edema induced by dimethylbenzene and vascular permeability induced by acetic acid. Furthermore, the MEAP could inhibit pain reflexes in response to chemical nociception induced by acetic acid and formalin. The results obtained in our present investigation also revealed that the mechanisms of anti-inflammatory and analgesic effects of MEAP may be related to the decreased expression levels of TNF-α, IL-6, iNOS and COX-2. However, further investigations are required to conclusively determine the bioactive compounds from MEAP.
